# Proteostasis and RNA Binding Proteins in Synaptic Plasticity and in the Pathogenesis of Neuropsychiatric Disorders

**DOI:** 10.1155/2016/3857934

**Published:** 2016-01-12

**Authors:** Matthew E. Klein, Hannah Monday, Bryen A. Jordan

**Affiliations:** ^1^Dominick P. Purpura Department of Neuroscience, Albert Einstein College of Medicine, 1300 Morris Park Avenue, Bronx, NY 10461, USA; ^2^Department of Psychiatry and Behavioral Sciences, Albert Einstein College of Medicine, 1300 Morris Park Avenue, Bronx, NY 10461, USA

## Abstract

Decades of research have demonstrated that rapid alterations in protein abundance are required for synaptic plasticity, a cellular correlate for learning and memory. Control of protein abundance, known as proteostasis, is achieved across a complex neuronal morphology that includes a tortuous axon as well as an extensive dendritic arbor supporting thousands of individual synaptic compartments. To regulate the spatiotemporal synthesis of proteins, neurons must efficiently coordinate the transport and metabolism of mRNAs. Among multiple levels of regulation, transacting RNA binding proteins (RBPs) control proteostasis by binding to mRNAs and mediating their transport and translation in response to synaptic activity. In addition to synthesis, protein degradation must be carefully balanced for optimal proteostasis, as deviations resulting in excess or insufficient abundance of key synaptic factors produce pathologies. As such, mutations in components of the proteasomal or translational machinery, including RBPs, have been linked to the pathogenesis of neurological disorders such as Fragile X Syndrome (FXS), Fragile X Tremor Ataxia Syndrome (FXTAS), and Autism Spectrum Disorders (ASD). In this review, we summarize recent scientific findings, highlight ongoing questions, and link basic molecular mechanisms to the pathogenesis of common neuropsychiatric disorders.

## 1. Ribonucleoproteins and RBPs

**Figure 1:**
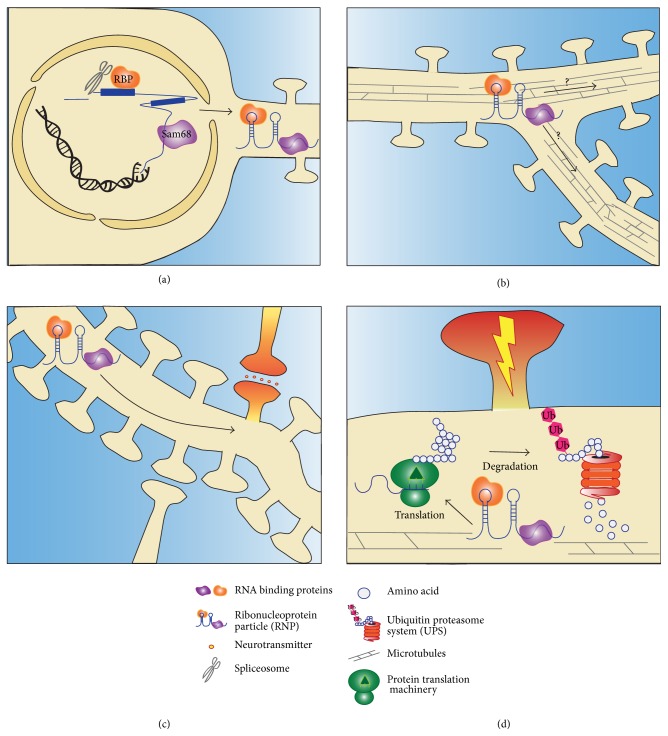
The trials and tribulations of RBPs and RNPs. (a) RBPs assemble cotranscriptionally and regulate mRNA splicing and modification preventing the coassembly of multiple mRNAs per RNP. Motifs in the 5′UTR and 3′UTR as well as retained intronic sequences facilitate dendritic targeting of RNPs. (b) RBPs transport mRNAs along microtubules to destinations dictated by the cargo mRNA sequence. Through input-specific events, synapses or dendritic branches may autonomously regulate their mRNA content. (c) Excitatory synapses at dendritic spines greatly outnumber mRNAs in dendrites and even more so counting inhibitory synapses. Despite being sparsely distributed, local mRNAs contribute significantly to synaptic function. (d) Upon synaptic stimulation, RBP function determines mRNA fate. Derepression by translational repressors can be followed by promotion of translation by RBPs like Sam68 (purple). Translation is counterbalanced by proteasomal or lysosomal degradation.

The majority of cytoplasmic mRNAs in neurons are associated with RBPs and other accessory proteins as part of large macromolecular complexes termed ribonucleoprotein (RNP) granules [1]. Granules are diverse in composition and function, mediating many aspects of posttranscriptional RNA regulation including cellular transport, protection from nucleases, and translational control [2–4]. The modular domain structure of RBPs facilitates granule formation and enables auxiliary interactions with other factors necessary to ensure precise localization and metabolism of mRNA cargos [5]. Recent evidence suggests that only one mRNA is present per RNP granule [6–9]. This stoichiometry may be achieved through cotranscriptional packaging of the RNP complex as many RBPs contain nuclear localization sequences and undergo nucleocytoplasmic transport [10, 11]. Following transcription, RBPs are thought to spontaneously couple to mRNA targets to facilitate processing of pre-mRNAs through splicing, editing, polyadenylation, and granule formation (Figure 1). In addition, many RBPs contain prion-like low complexity sequences that may allow for spontaneous self-aggregation. Thus, cargo mRNAs could act as scaffolds/nucleators for aggregation by recruiting and increasing the local concentration of RBPs to catalyze spontaneous granule formation [12]. Low complexity sequences may also promote heteroaggregation of accessory proteins into granules and allow for the dynamic disassembly and reformation of RNP granules in response to synaptic activity [7, 12, 13].

## 2. RBPs and Neuronal Dendritic Targeting

The control of RNA distribution is a fundamental mechanism underlying localized expression of proteins [14]. This is especially pertinent among the intricate dendritic arbors of neurons where synapses are thought to independently alter their morphology and function in response to neuronal activity via spatial restriction of gene expression. In addition to diffusion and transport of somatically generated proteins, synthesis from dendritically targeted mRNAs shapes the local proteome around synaptic contacts. The active transport of mRNAs into neuronal processes requires their association with RNA transport particles, which contain (1) specific RBPs to prevent translation prior to delivery, (2) adaptors for association with cytoskeletal translocation machinery, and (3) molecular motors [15] (Figure 1). A number of groups have identified dendritic targeting elements (DTEs) in the 3′ untranslated regions (3′UTR) of mRNAs present in synaptodendritic compartments including CaMKII*α*, beta actin, MAP2, ARC, and BDNF [16–24]. BDNF and CaMKII*α* isoforms with shortened 3′UTRs lacking DTEs are not dendritically targeted, suggesting that alternative splicing of cis-elements can regulate mRNA localization [16, 25]. One of the best-studied DTEs is the “zipcode” found in the 3′UTR of beta-actin mRNA, which is recognized by the RBP Zipcode Binding Protein 1 (ZBP1) and is necessary for its transport and translational regulation [19]. Interestingly, the beta-actin 3′UTR contains an additional nonoverlapping DTE recognized by the RBP Src-Associated in Mitosis 68 KDa (Sam68) [26]. We have shown that Sam68 is crucial for the dendritic transport and translation of beta-actin mRNA, similar to ZBP1 [27]. Whether multiple DTEs allow for concurrent binding of RBPs is an outstanding question in the field and will be addressed in more detail later in this review.

Diverse mRNA targeting mechanisms have been identified in neurons. Retained introns in cytoplasmic mRNAs represent an additional and surprising class of cis-acting dendritic targeting elements that are regulated by the spliceosome. Cytoplasmic intron-sequence retaining transcripts (CIRTs) contain intronic elements that are sufficient to target these mRNAs to dendrites [28–30]. The presence of spliceosome constituents in dendrites raises the intriguing possibility that localized splicing may represent a previously unappreciated activity-dependent cytoplasmic process [31]. In this manner, incompletely processed CIRTs could be transitionally repressed until fully spliced and made competent by the dendritic spliceosome [32]. Indeed, several RBPs implicated in splicing have been observed in synaptodendritic compartments [11, 33] including Sam68 [34–38].

Many of the over 2550 mRNAs present in dendrites [39, 40] lack any known dendritic targeting element, suggesting the existence of an alternative targeting mechanism. Recent evidence implicates nonsequence specific, structural elements in both the 3′UTRs and 5′UTRs in mRNAs in dendritic and synaptic localization [23]. Structural G-quartet stem loops, rather than sequence recognition, have been implicated in how the RBP Fragile X Mental Retardation Protein (FMRP) binds to mRNA cargos [41–43]. However, recent additional evidence indicates binding sites may be present throughout the entire sequence [44] or at specific 3′UTR sites [45]. Altogether, these findings suggest the existence of functionally distinct mechanisms to localize RNAs within neuronal processes and highlight the role of noncoding mRNA sequences in subcellular targeting. Recent genome-wide association studies suggest that mutations in noncoding targeting elements are linked with disease phenotypes, underscoring the importance of mRNA transport in supporting neuronal function [46, 47].

## 3. RBPs and Input-Specific Translation

Input specificity requires the localized expression of proteins following mRNA transport. Dendritic translation in neurons was first evidenced by the presence of ribosomes [48, 49] and mRNAs [50, 51] closely associated with postsynaptic contacts. This was later confirmed by studies demonstrating that mechanically isolated dendrites are translation-competent [24, 52] and can sustain protein synthesis-dependent forms of synaptic plasticity [24, 53, 54]. Furthermore, the transport and localized translation of mRNAs are both synapse- and stimulus-specific [24].

RBPs like FMRP and ZBP1 mediate translational repression and release cargos locally in response to particular stimulus-driven posttranslational modifications. For example, locally active Src kinase phosphorylates ZBP1 upon arrival at the synapse, which reduces its affinity for beta-actin mRNA and liberates it for subsequent translation [55]. Other RBPs are thought to directly promote protein synthesis. Sam68, for example, has been recently shown to positively regulate translation in neurons [34, 36, 37, 56–58]. Our group found that Sam68 regulates the levels of beta-actin mRNA and protein in synaptodendritic compartments [27]. We found that Sam68 regulates the loading of beta-actin mRNA onto polysomes isolated from synaptic fractions, suggesting that Sam68 promotes local protein synthesis. Sam68 was originally identified as Src binding partner [59] and phosphorylation of Sam68 by both tyrosine and serine-threonine kinases has been shown to regulate its affinity for RNA [60–62]. Therefore, synaptic activity may trigger downstream signaling cascades that affect local Sam68-dependent protein synthesis. New research aimed at disentangling the molecular mechanisms that regulate the spatial and temporal derepression and translation of mRNAs will help to further our understanding of RBPs in synaptic function and brain disorders.

## 4. Balancing Protein Synthesis and Degradation in Synaptic Plasticity and Disease

Protein translation and degradation both contribute to proteostasis and are essential for proper synaptic function. Aberrant protein levels at synapses are thought to be pathogenic primarily by affecting the expression and/or maintenance of synaptic transmission and plasticity. Several ASD susceptibility genes encode for proteins that regulate translation, or proteasomal degradation (outlined below). These genes are also involved in the induction and expression of mGluR-mediated long-term depression (mGluR-LTD), a type of synaptic plasticity that requires protein synthesis [63–65]. Deficits in mGluR-LTD have been observed in numerous mouse models of ASDs and other cognitive and neurodegenerative disorders such as Alzheimer's and Parkinson's disease [65–70]. One of the most widely studied examples is that of the translational repressor FMRP [44], whose absence leads to Fragile X Syndrome (FXS), the most common form of inherited intellectual disability in boys. FMRP binds to a plethora of mRNAs suggesting that the underlying pathology of FXS may result from broad translational dysregulation of the neuronal transcriptome [44]. Mice null for FMRP display general increases in basal protein synthesis [71] but lack translation in response to activation of mGluRs [72]. Paradoxically, FMRP KO mice exhibit exaggerated mGluR-LTD despite a lack of mGluR-triggered protein synthesis. One theory is that elevated basal levels of plasticity-related proteins in the FMRP null mice bypass the need for mGluR-triggered translation, thus resulting in enhanced mGluR-LTD, which may contribute to the neurological symptoms of FXS [73]. However, recent work from our group demonstrates that the magnitude of mGluR-LTD is not necessarily correlated with synaptic protein abundance [56]. Other deficits, including altered neuronal excitability [74–76] or decreased proteasome function [77, 78], could underlie the exaggerated LTD observed in FMRP null mice.

In addition to FXS and ASD, RBP dysfunction may also play a role in other disorders including schizophrenia and amyotrophic lateral sclerosis. The protein disrupted in schizophrenia-1 (DISC-1) [79] was recently identified as a novel RBP and a component of RNP granules [80]. Unregulated expression of DISC-1 has been associated with schizophrenia and clinical depression. DISC-1 appears to be important for dendritic mRNA transport and for maintenance of late phase long-term potentiation (L-LTP) [80]. Accumulation of the transactive response DNA-binding protein-43 (TDP-43) in the cytoplasm is evident in sporadic forms of amyotrophic lateral sclerosis, frontotemporal dementia, and Alzheimer's disease [81]. TDP-43 binds to DNA and RNA and has been shown to regulate splicing, mRNA stability, and mRNA transport and translation as well as synaptic function in motor neurons [82, 83]. These results suggest a broad role for RBP in normal and pathological brain function.

Consistent with links between unbalanced protein levels and disease, several neuropsychiatric disorders have been associated with mutations in components of the ubiquitin proteasome system (UPS) [84–86] including Parkinson's disease, spinocerebellar ataxia, X-Linked Mental Retardation, and Angelman Syndrome [87–92]. Evidence for transport of proteasomal subunits and E3 ligases into dendritic spines [93] and activity-dependent ubiquitination of the synaptic proteome [94, 95] suggests that local effects of the UPS contribute to synaptic proteostasis and input specificity. Since the UPS degrades proteins, one might expect that pathologies associated with impaired UPS function would arise from a toxic accumulation of substrates. However, this is not always the case, suggesting that UPS function is more complex than initially imagined [96]. For example, monoubiquitination of diverse synaptic proteins can regulate synaptic transmission independent of protein degradation [97, 98]. Nonproteolytic monoubiquitination of the RBP CPEB3 regulates dendritic spine growth and AMPA receptor abundance and ultimately regulates learning and memory [99]. Moreover, monoubiquitination of PSD95 [100, 101] and PICK1 by Parkin [102] may regulate the surface expression of AMPA subunits and the acid sensing channel, respectively. Several groups have demonstrated acute inhibition of the proteasome affects long-term plasticity, but there is some disagreement in the literature on the nature of the disruptions [56, 103–106].

In the case of FXS, studies demonstrate that FMRP is rapidly degraded by the proteasome during the induction of synaptic plasticity [105, 107]. Therefore, mutations that inhibit proteasomal degradation of FMRP may lead to altered plasticity and result in neuropsychiatric symptoms. Consistent with this hypothesis, a recent study discovered that loss of the E3 ubiquitin ligase Cdh1-APC prevents FMRP degradation, as well as the induction of mGluR-LTD [108]. Furthermore, previous work in the Cdh1 knockout mouse identified a deficit in late phase LTP and contextual fear conditioning [109]. Therefore, the interaction between FMRP and Cdh1-APC may be essential for multiple forms of plasticity and memory formation. Presumably, degradation of FMRP would positively impact the translation of its cargo mRNAs to support long-term postsynaptic changes, though the direct effect of FMRP degradation on protein levels remains to be addressed.

Fragile X Tremor Ataxia Syndrome (FXTAS) is a neurodegenerative disorder characterized by adult-onset ataxia and cognitive decline. In FXTAS, a pathogenic premutation trinucleotide repeat expansion (50–200 repeats) in the 5′UTR of the FMR1 gene (FMRP) does not result in transcriptional repression but rather causes a toxic gain-of-function through the formation of intranuclear inclusions containing the FMR1 mRNA and sequestered RBPs [110]. Recent work suggests that the sequestration of crucial cellular factors, including Sam68, into these intranuclear inclusions contributes to the cognitive deficits observed in FXTAS [111]. Indeed, Sam68 is functionally impaired in FXTAS patient tissue [111] and accumulation at intranuclear inclusions precedes other deficits, suggesting that loss of Sam68 function plays a causal role in FXTAS [111]. Sam68 KO mice display ataxia [57, 112] and both Sam68 KO mice and primary neurons lacking Sam68 display deficits in dendritic spine morphology [27], which is also seen in other FXTAS models using expanded CGG repeats [113].

We have recently shown that Sam68 is critically involved in coordinating mRNA translation and degradation via the proteasome during the induction of synaptic plasticity. Sam68 is likely necessary to promote the rapid translation of several plasticity-related proteins in response to mGluR activation. In Sam68 KO animals, the balance of proteostasis is abnormal and tipped towards degradation. Interestingly, Sam68 KO mice display impaired mGluR-LTD that can be rescued by blocking the proteasome [56]. In our model, rapid proteasomal degradation acts as a homeostatic scaling mechanism to prevent the accumulation of plasticity-related proteins and thus the induction of further rounds of mGluR-LTD, independent of mGluR activation. Our recent research has put together a more nuanced view of synaptic proteostasis in synaptic plasticity, as a push-pull between RBP-mediated translation and proteasomal degradation. Disruptions to this balance may underlie the pathogenesis of neuropsychiatric disorders including FXTAS.

## 5. Unanswered Questions

### 5.1. Is There Presynaptic Protein Synthesis?

In accordance with current scientific research, the bulk of this review has focused on the mechanisms by which RBPs regulate* post*synaptic sites. However, many forms of synaptic plasticity are presynaptically expressed in vertebrate and invertebrate preparations and may require protein synthesis [114–116]. Over 300 mRNA species identified in mature axons include transcripts encoding for components of the translation machinery [117]. During development, the RBPs ZBP1, FMRP, and CPEB respond to stimulus-specific cues in axonal growth cones to mediate mRNA translation [41, 118–121] and ZBP1 localizes at axonal branch points where it mediates beta-actin mRNA translation and branch stabilization [122]. In mature axons, it was recently shown that amyloid beta peptides stimulate the axonal synthesis of the transcription factor ATF4 among other proteins, which can shuttle into the presynaptic nucleus and initiate cell death [123]. It has been postulated that axons use a different type of translation machinery (e.g. monosomes v. polysomes) [124, 125] and that ribosomes are localized to electron-dense regions and/or tethered to the cell membrane within the axon [125, 126]. This may explain why structural evidence for presynaptic/axonal ribosomes is scarce. As many neuronal subtypes have highly branched axonal projections that synapse on multiple neurons, one would also expect input-specific regulation of presynaptic function. RBPs provide a plausible mechanism by which this regulation could be accomplished in adult CNS axons. The development and implementation of new strategies to isolate and visualize axons and presynaptic compartments should inform this line of study.

### 5.2. How Is Specificity Achieved Given the Ratio of Synapses to RNAs?

The breadth of the dendritic transcriptome supports an important role for local translation in long-term plasticity [39]. However, the mRNAs for many important synaptic proteins such as BDNF, GluA2, SHANK, and ARC are conspicuous in their scarcity or absence in the dendrite [6, 9, 13, 23]. Furthermore, the number of even the most abundant dendritic mRNAs (beta-actin and CaMKII*α*) is typically an order of magnitude less than the number of synapses. This discrepancy poses the simple mechanistic problem that there are not nearly enough mRNAs to supply proteins on a one to one basis with synapses as required. This problem may be circumvented by a high translational efficiency of synaptic mRNAs, with each mRNA being translated many times to produce an adequate number of proteins. Newly synthesized proteins would then traffic towards the appropriate synapse and thus few mRNAs could supply proteins to an entire dendritic branch, rather than a single dendritic spine or synapse. To our knowledge, direct measurements of mRNA translational efficiency at synapses have been prohibitively difficult to obtain. Exciting new developments in fluorescent tagging, including spaghetti monster fluorescent proteins [127] and SunTag [128], may soon allow for the direct visualization and measurement of local translation.

Additionally, electron microscopy studies in adult hippocampus reveal that there are far fewer dendritic polysomes (the presumed sites of local translation) than synapses [129–131]. Perhaps only a subset of synapses undergo long-term morphological and functional changes or require protein synthesis to do so. Large dendritic spines containing the spine apparatus and endoplasmic reticulum [132] could comprise this group. Alternatively, the rapid and bidirectional transport of mRNAs by RBPs towards active synapses along neuronal dendrites may be an ongoing process long after transcription [133]. In this case, synapses undergoing plasticity might physically capture transporting mRNP granules through unknown mechanisms. Indeed, a portion of dendritic beta-actin mRNAs display active and bidirectional transport [134] and polysomes themselves can redistribute from dendritic shafts into spines in response to a plasticity-inducing stimulus [135].

As we propose in the previous paragraphs, the discovery of motile dendritic RNPs suggests that the local area served by a single mRNA may well be a dendritic branch rather than a single spine. Recent work suggests CaMKII*α* mRNA and protein demonstrate branch specificity in response to mTOR activity [136]. If the dendritic branch rather than individual synapses represents the consolidated integrative unit underlying translation-dependent forms of plasticity as previously suggested [137, 138], then input specificity might refer to a branch, rather than an individual synaptic junction. Under these conditions, having few dynamically transported mRNAs at each dendritic branch may be sufficient for plasticity. New massively multiplexed, FISH-based techniques to localize all the mRNAs in a neuron will allow for the determination of the spatial relationship of mRNAs to synapses and branch points [139, 140]. In concert with these new techniques, further experiments using more physiological inductions of plasticity along with mRNA visualization will be of great benefit in elucidating the movements of dendritic mRNAs and the spatial extent of “local” translation.

### 5.3. What Is the Contribution of Locally Translated Protein to the Existing Local Pool?

To our knowledge, accurate numbers of actin molecules at neuronal dendritic spines have not been calculated and likely vary substantially based on conditions. However, an estimate based on studies of stereocilia of the inner ear [141], which are actin-rich protrusions of similar size, suggests 10^5^ actin molecules per spine (each stereocilia contains ~100–700 actin filaments on average and 370 actin molecules per micron of actin filament). Considering ribosomal processing speeds (6–9 amino acids/sec) [142] and the size of beta actin, we estimate that one beta-actin protein can be produced every 50 seconds, or 36 beta-actin molecules can be produced in 30 minutes per mRNA and per ribosome. Even considering multiple mRNAs and polyribosomes, the amount of newly synthesized beta-actin would likely represent only a small fraction of available synaptic molecules. If the transport and translation of beta-actin mRNAs into dendritic spines contribute to morphological plasticity following synaptic stimulation, then a rationale must be found for why this population must be* newly* made as opposed to being recycled from large synaptic pools or transported from the cell body. Temporally regulated irreversible posttranslational modifications may functionally distinguish beta-actin molecules. Indeed, newly synthesized beta-actin localizes at the leading edge of filament formation, perhaps through fast arginylation at the N-terminus [143], which has been previously shown to increase actin polymerization [144, 145]. In addition, spatially regulated posttranslational modifications may also confer functional distinctions on newly synthesized proteins. BDNF synthesized at dendrites has been implicated in spine head growth and pruning, whereas BDNF synthesized in the cell body promotes spine formation [146]. Thus, though the contribution of local translation to total dendritic protein for highly abundant proteins may be small, the functional distinction of newly and locally synthesized proteins may be the primary effector of synaptic alterations. The recent development of techniques that enable the visualization and quantification of newly synthesized proteins, such as fluorescent noncanonical amino acid tagging (FUNCAT) [147], may provide new insight into the contribution of RBP-mediated local translation to the total protein pool.

### 5.4. Do Multiple RBPs Bind to RNAs?

Several mRNAs contain nonoverlapping binding sites for diverse RBPs, suggesting complex and multifactorial regulation of mRNA metabolism. Beta-actin mRNA itself contains nonoverlapping binding sites for ZBP1 [19], Sam68 [26], and FMRP [45], although how these RBPs combine to regulate beta-actin metabolism is unknown. We compared Sam68 mRNA cargos identified using UV-crosslinking techniques [36] and found that 83.7% of these mRNAs also bound to FMRP [44, 45]. As loss of protein synthesis* promoted* by Sam68 leads to* impaired* mGluR-LTD and loss of FMRP* repression* leads to* enhanced* mGluR-LTD [148], Sam68 and FMRP could bind cooperatively to bidirectionally regulate RNA cargo metabolism. Thus, Sam68 and FMRP may differentially regulate a common pool of dendritically expressed neuronal mRNAs to regulate synaptic function, although whether they bind at the same time is unclear. It is interesting to speculate how the opposing actions of these RBPs coordinate the metabolism of single mRNA. Perhaps diverse RBPs regulate stimulus-specific synaptic activity. In this way, a different translational response could be activated after a weak or strong stimulus, or from different types of synaptic activity (i.e., excitatory versus inhibitory, metabotropic versus ionotropic). Perhaps multi-RBP regulation of mRNAs provides additional layers of regulation for fine-tuning spatial and temporal protein expression. Answers to these questions remain unclear and will require additional experimentation.

## 6. Conclusion

To achieve proteostasis, neurons must spatially coordinate multiple cellular processes across thousands of synapses. In the cellular milieu, mRNAs are always packaged into ribonucleoprotein (RNP) granules, which coordinate the transport and translation of their cargo mRNAs. Disease causing mutations in mRNAs or RBPs may lead to disruption of mRNA packaging into granules and alter subsequent transport and translation. These pathological mechanisms underscore the importance of mRNA being in the right place at the right time, as well as the importance of mRNA as a structural platform to coordinate interactions between RBPs and associated proteins. However, many unanswered questions remain, including a lack of sufficient mRNA particles for synapses, the contribution of local translation to existing pools of protein, and the interactions and complex regulation of multiple RBPs per single mRNA. There are numerous limitations in studying mRNA trafficking and translation in neurons, such as the lack of an assay to determine the exact localization and timing of synthesized proteins (however, see [149]). Moreover, most transcriptional and mRNA transport processes have been studied in the context of strong and nonphysiological stimuli, such as bath application of neurotransmitters in cellular cultures. As techniques for single synapse stimulation and single molecule imaging of mRNA in live tissue improve, we may observe different behavior of mRNA transport under more physiological stimulation paradigms.
